# Social-Cultural Processes and Urban Affordances for Healthy and Sustainable Food Consumption

**DOI:** 10.3389/fpsyg.2018.02407

**Published:** 2018-12-06

**Authors:** Giuseppe Carrus, Sabine Pirchio, Stefano Mastandrea

**Affiliations:** ^1^Department of Education, Experimental Psychology Laboratory, Roma Tre University, Rome, Italy; ^2^Department of Dynamic and Clinical Psychology, Sapienza University of Rome, Rome, Italy

**Keywords:** social norms, food consumption, cultural processes, sustainability, wellbeing, restorative environments, walkability, urban affordances

## Abstract

In this paper, we provide an overview of research highlighting the relation between cultural processes, social norms, and food choices, discussing the implication of these findings for the promotion of more sustainable lifestyles. Our aim is to outline how environmental psychological research on urban affordances, through the specific concepts of restorative environments and walkability, could complement these findings to better understand human health, wellbeing and quality of life. We highlight how social norms and cultural processes are linked to food choices, and we discuss the possible health-related outcomes of cultural differences in food practices as well as their relation to acculturation and globalization processes. We also discuss the concepts of restorative environments and walkability as positive urban affordances, their relation to human wellbeing, and the possible link with cultural processes and sustainable lifestyles. Finally, we outline issues for future research and areas for policy-making and interventions on the links between cultural processes, healthy and sustainable food consumption and urban affordances, for the pursuit of public health, wellbeing and environmental sustainability.

## Introduction

The multi-cultural reality created by the worldwide phenomena of globalization, immigration, mass media communication and global social networking points to the need of deeply understanding the cultural and sub-cultural realities to comprehend individual behavioral change within any given country (Shweder and Sullivan, 1993; Stokols, 2018). The growing importance of the migratory phenomenon on the global level (for statistical facts on this issue see United Nations, 2017) over the last decades and the related social, political, economic and educational implications (see for example Pirchio et al., 2015, 2017a,b, 2018; Passiatore et al., 2017 for a discussion of these issues in the field of multicultural education and language learning) suggests also the relevance of ethnic identities in modern societies for understanding sustainable lifestyle change. Among the cultural dimensions connected to ethnicity, food preferences, preparation methods, and consumption choices have a central role, with relevant implications in terms of social and environmental sustainability. Research in anthropology has shown that food is a key expression of human cultures and subcultures. Far from being only a means of survival, the relation between human beings and food in modern societies has rather to be considered for its symbolic value, connected to the development of the individual, social and collective self (Cleveland et al., 2009).

In this paper, we discuss the issue of cultural processes in food consumption, its implication for human wellbeing and environmental sustainability, and its relations to two distinct features of urban affordances, namely restorative environments and walkability. The cultural dimension of urban affordances, walkability and restorative environments could be an interesting concept for bridging together different theoretical approaches and historic roots in environmental psychology. While the notion of affordances can be traced back to Gibson’s (2014) ecological approach to perception (see also Heft, 2010), restorative environments have typically been studied in relation to cognitive processes and human health (e.g., Ulrich, 1983; Kaplan and Kaplan, 1989; Hartig, 2004) or social psychology (e.g., Staats and Hartig, 2004). Interestingly, the links between cultural processes and affordances have been previously investigated in studies on child development (e.g., Kyttä, 2002, 2004), and place-based pedagogies has also been used to engage refugee students re-settling in Australia (e.g., Comber and Nixon, 2013).

## Social Influence and Cultural Norms in Food Consumption and Dietary Choices

Food choices and dietary patterns (e.g., food preferences, preparation methods, consumption) represent a central aspect at the basis of human social and cultural diversity (Fischler, 1988). These ideas have long been in the focus of classical anthropological research on food habits and eating preferences (e.g., Harris, 1998). Many other scholars have highlighted in a similar way the role of food preparation and consumption for the development of social identity, among people with an immigrant background or other minority groups, and the implications of this for cultural adaptation processes (e.g., Oswald, 1999; Gvion, 2009). Indeed, for many human societies and groups in a condition of immigration, food preparation and consumption often constitute a way for complementing traditional elements inherited from the home country with new ones derived from the host society. A review of the literature in this field, which we discuss in this section, taking into account the social, health and economic implications, allowed us to outline the following main aspects:

- variations in intake patterns and food practices occur across different ethnic groups, and are subject to mutual influence (e.g., Rozin et al., 1999);
- (un)healthy and (un)sustainable intake patterns and food practices can be typical of different ethnic groups, but they also could be challenged through educational programs, cultural transitions, lifestyle change public policies, and urban planning interventions (e.g., Gittelsohn and Vastine, 2003).

An opportunity to observe the cultural categorization process as it applies to food emerges from looking at how eating and dieting patterns develop and change in multicultural environments. This aspect is relevant for health promotion, although traditionally the objective content of nutrients in food, rather than attitudes toward food and food-related lifestyles, has been prioritized for understanding health issues and longevity. The phenomenon known as the “French Paradox” offers a pertinent example of this issue (Renaud and de Lorgeril, 1992). The French paradox identifies the weak link between saturated fat intake and the incidence of heart disease recorded by epidemiological studies in the French population compared to other populations (American or Japanese, for instance). A cultural perspective may then shed light to better understand the clinical facts, as it allows one to identify differences across countries that are likely to account for the paradox (e.g., Ferrières, 2004). In fact, the answers to the paradox should be mostly searched for in objective and subjective protective factors such as cultural norms, values and lifestyles, stressful versus enjoyable experience of food intake, levels of physical activity in the population, rather than looking at possible causes in genetics (Rozin et al., 1999). Therefore, the consideration of the social resources and constraints in defining dietary choices leads to a definition of health that corresponds not only to the absence of illness, but as part of a process involving the social, economic and environmental systems. In this perspective, health cannot be conceived without social and environmental sustainability (Kjærgård et al., 2014).

But what happens when different sets of cultural norms and identities meet in the same individual? Studies focusing on the experience of migrant people describe the changes in dietary habits and lifestyle associated to migration, and how this affects health status. Generally, it has been shown that migrants adopt the dietary patterns of the host country as part of their acculturation process. Acculturation is usually defined as a socio-psychological process of adaptation to a new cultural milieu, which is mediated by social, demographic and economic factors (such as gender, education or income) and by the development of individual lifestyles (Berry and Sam, 1997). While acculturation theoretically implies changes either in the individuals belonging to the minority group and in the host culture, nevertheless most of the literature focuses on the migrants’ changes due to the adaptation to their new country (Rogler et al., 1991).

Usually, members of specific ethnic groups in a given society experience complex patterns of adaptation, based on the interplay between the acquisition of specific skills to function within a host culture and the conservation of features related to the home-country cultures. Sometimes a change in dietary pattern toward the adoption of the host country habits may be detrimental for migrants’ health when the home country’s lifestyle is healthier than the new country’s one (e.g., Cardoso et al., 1997; Anderson et al., 2005). Sometimes, on the contrary, the adoption of the new country’s lifestyle is accompanied by the maintenance of traditional eating habits, especially at the beginning of the acculturation process, or in conditions where the exposure to the home country cultural norms is assured. The increase in processed and fat food consumption, and the consequent increase in body weight and related diseases as hypertension, diabetes and cardiovascular disorders, is also a common outcome of the societal transformation from a rural and traditional culture to the contemporary urban industrialized culture. This is the case, for example, of many sub-Saharan African countries (e.g., Walker, 1964; Holdsworth et al., 2004).

Works reviewed so far thus suggest that in many situations of cultural change and migration, preserving and improving food traditions from the home country and supporting a lifestyle that includes sufficient physical activity should be considered as a fundamental goal for health promotion programs and education interventions (e.g., Méjean et al., 2009; Satia, 2010; Popovic-Lipovac and Strasser, 2015).

The aspect here discussed not only interact with health promotion in food consumption but may also play a role in sustainable lifestyles. Health and sustainability are with no doubt linked in the field of food production and consumption (Johnston et al., 2014), but this link often fails to be translated in a fully integrative perspective while developing local or global policies. This dual perspective would imply that both health and sustainability should be considered as important issues by policymakers and other relevant stakeholders. Prevention authorities might thus want to keep both of them in mind when planning and implementing policies and developing strategies in the public health domain: without such a perspective, the likelihood and risks of negative social, environmental or health consequences may increase (Kjærgård et al., 2014). In fact, environmental, social and economic features of life contexts (e.g., the unconditional availability of food, in particular in the form of fast, high-sugar, and high-fat and fast foods) may lead to individual behaviors, such as uncontrolled or impulsive consumption, which are, in turn, detrimental not only for the individual health (e.g., obesity and its many comorbidities) but also affect economic and environmental sustainability, as stated by major intergovernmental bodies (e.g., Gonzalez Fischer and Garnett, 2016; see also Drewnowski, 2014). The unnecessary overproduction or waste of food, to meet unnecessary overconsumption demands in affluent societies, contributes in fact to the depletion of the planet’s resources, such as the soil, air and fossil fuels, and negatively impacts on the wellbeing of current and future generations (Reisch and Gwozdz, 2011).

## Culture, Dietary Patterns and Sustainability

A major trend in (un)healthy and (un)sustainable food consumption and production seems to be occurring at the global level, across different cultural contexts (see^1^ for a more detailed discussion and global trends on this issue). The food production and consumption systems have serious and intertwined implications for both public health and environmental quality, worldwide. For example, livestock food production is a substantial source of greenhouse -gas emissions and a source of some essential nutrients, but at the same time meat provides large amounts of saturated fat, which is a known risk factor for cardiovascular disease (e.g., Garnett, 2009). In particular, issues that have been identified as crucial for understanding the relation among food choices, cultural processes and sustainability refer to factors such as the public health benefits of greenhouse gas reduction policies in the domain of food habits and consumption (e.g., Friel et al., 2009), to the adaption of food systems and food security in relation to global environmental changes (e.g., Ericksen, 2008), and to the links between globalization, dietary patterns and nutrition transitions in different areas of the world (particularly middle-income countries; e.g., Hawkes, 2006).

Higher intake of fruits and vegetables is usually associated with many health benefits, such as decreased risk of diabetes and metabolic syndromes, heart disease, and cancer, and is at the same time associated with a lower carbon footprint. Thus, dietary shifts away from meat and dairy products are recommendable for both public health and environmental reasons (e.g., Friel et al., 2009; Garnett, 2011). In this perspective, many studies analyzed the role of ethnicity in shaping food choices and consumption in western societies, by referring to the already mentioned process of acculturation. In fact, the analysis of the migrants’ changes in food consumption may clarify the factors and variables fostering and limiting healthy and sustainable lifestyles. Studies that investigated the food intake patterns of groups with Hispanic origins, living in United States or Canada (e.g., Bermudez et al., 2000) seem to suggest for example that the acculturation process might be associated with lower fiber-rich food and higher added-sugar food intake. The transition to more North American dietary styles is often driven by the difficulty of finding food products from the original cultures, as well as fresh fruit and vegetables (Satia et al., 2000). Major barriers to healthy food choices among immigrant groups in North America seem thus to be related to the high cost of fruit and vegetables, to the lower energy supply and higher preparation time required for eating fruit and vegetables, and to the negative impact of mass media advertising; it is harder to see fruit and vegetables than unhealthy food in TV ads in TV (e.g., Klepp et al., 2007), but might also be linked to specific spatial features of everyday living environments.

Indeed, cultural or country differences might not fully account for the variations in diet styles, food intake and health outcomes that many studies outline. In fact, centrally to the purpose of this paper, we propose that affordances from the physical environment could also be a relevant factor deserving systematic investigation in order to understand food consumption and the related health outcomes across different cultures and subcultures in contemporary cities. We argue here that features and affordances of the physical context, such as for example the presence of opportunities for psychological restoration in natural settings in the city (e.g., Hartig, 2004), or the supportiveness of outdoor environments (e.g., Curl et al., 2016), could also play a relevant role in shaping our habits in relation to food consumption or other health-related habits. Likewise, it has been suggested that a specific feature like walkability could be related to human health and wellbeing in present-day urban settings, also through increased physical activity (e.g., Brown et al., 2013; Van Cauwenberg et al., 2016). We argue that walkability and physical activity could also be influential, by working in synergy with restorative environments, and help the transition toward healthier and more sustainable lifestyles (e.g., Rioux et al., 2016). For example, we could speculate that urban planning solutions that afford walking and promote nature-based outdoor activities could also have a positive impact on promoting social inclusion and intergroup contact, thus reducing interpersonal distances across different cultural or ethnic groups (e.g., de Vries et al., 2013). Food lifestyle could also be beneficially impacted in this case, as healthier and more sustainable food purchases might also be more likely if adequately supported by positive urban affordances. Indeed, many quotidian settings, where individuals are actively present and intellectually engaged in evaluating their surroundings (e.g., Alves, 2014; see also Mercado-Doménech et al., 2017), could be assumed to have beneficial outcomes on human health and wellbeing, as shown in a recent study by Mastandrea et al. (2018) on the restorative effects after visions of figurative and abstract art paintings in art museums (see also Chirumbolo et al., 2014 and Mastandrea and Crano, 2018 for more detailed accounts on the social cognitive mechanisms of art preferences).

## Restorative Environments and Walkability as Urban Affordances for Health Promotion and More Sustainable Food Consumption

Restorative environments are usually referred to as those settings that promote, and not simply permit, the experience of the recovery of a general psychological condition of wellbeing, the reduction of stress, the recovery of direct attention, and the increase of positive emotions (Ulrich, 1983; Kaplan and Kaplan, 1989; Hartig, 2004; Berman et al., 2008). More recent works also suggests that experiencing restoration opportunities may also positively affect social relationships in adults, families, and children (e.g., Taylor et al., 2002; Guéguen and Stefan, 2014; Carrus et al., 2015a, 2017; Hipp et al., 2016; see also Korpela et al., 2018). The literature on this subject has shown how natural environments, such as for example urban parks, peri-urban green areas, or extra urban nature preserves generally possess a high restorative potential (e.g., Hartig et al., 2011; Carrus et al., 2015b).

But how can urban restorative environments, in particular green spaces in compact cities, be related to the issue of a more sustainable and healthier food choices and lifestyles? Speaking about affordances, a link between these two aspects can be established through the issue of walkability. The concept of walkability has been recenly proposed to identify those physical and social characteristics of life contexts that promote pedestrian mobility and thus induce greater exercise and physical activity (e.g., Saelens et al., 2003; Brown et al., 2007). Walkability seems to be a function of several environmental factors, both at a “macro” level (e.g., density and connectivity of the roads, proximity of services) and at a “micro” level (e.g., aesthetic pleasure of places, perceived security, presence of green areas and sidewalks). In general, different authors have suggested a clear link between the walkability of residential environments and public health and wellbeing (e.g., Saelens et al., 2003; Brown et al., 2007; Brown and Werner, 2012). Likewise, access to outdoor green space and the possibility of being physically active in the residentials setting are important predictors of quality of life in urban settings (see Pol et al., 2017). According to Brown and Werner (2012), walking is a specific kind of daily life everyday physical activity and an important component for the promotion of public health. These authors refer to walkability also as a tool for contrasting the so-called obesity epidemic of affluent industrialized human societies. A decreasing trend in the physical activity level is in fact characterizing the present unsustainable lifestyle of large strata of the western population, also because individualized dieting and exercise programs implemented within public health interventions have not always succeeded in reaching their targets (see for example Catenacci et al., 2009). Density, street connectivity and proximity to stores were identified by meta-analytical studies as macro-level predictors of walkability (e.g., Saelens and Handy, 2008; Ewing and Cervero, 2010), while neighborhood green spaces, neighborhood aesthetics, perceived safety and social support have been associated with walkability at a micro-level (Brown and Werner, 2012). Thus, we could assume that more walkable urban settings should also be more restorative, with positive consequences for human wellbeing (e.g., Sugiyama et al., 2009), although more research is needed around this issue. Most importantly, providing walking- friendly structures in contemporary urban settings can be a relatively easy and cost-effective way to promote physical activity and pedestrian mobility, and more in general to initiate processes of lifestyle change and stimulate transitions toward sustainability at an individual and community level. What can be the connections between urban affordances such as restorative environments and walkable paths, on the one hand, and a more healthier and more sustainable lifestyle in the domain of food consumption, on the other hand? An argument for such a link is offered by recent studies that have explored the possible associations between the availability of healthy food stores, neighborhood walkability and general health indexes at the population level. A work by Rundle et al. (2008), showed, for example, how access to BMI-healthy food stores is associated with lower BMI and a lower prevalence of obesity among adult residents of New York City, also controlling for neighborhood walkability. At the same time, other works have explored the link between walkability and restorative environments, showing how the presence of green spaces can increase walkability (e.g., Rioux et al., 2016), and suggesting how public open space with affordances for physical activity could be an important source for health promotion at the community level (e.g., Giles-Corti et al., 2005). Stimulating people to spend more time outside as well as being physically active and walking, could then be a tools for shifting toward more balanced, healthier, and more sustainable food consumption patterns. A systematic review on green space and obesity suggested indeed that green space could be a positive factor to tackle weight-related health problems, although evidence in this field is not yet robust and abundant (Lachowycz and Jones, 2011). The presence of urban affordances, such as urban green spaces and walkable streets that increase the likelihood of exposure to healthy food stores, might be crucial in this sense (e.g., Paquet et al., 2017). A key mechanism could also be the possibility of experiencing psychological restoration in urban natural settings and a deeper sense of connectedness to nature, which seems to be associated with a better higher capacity of for self-regulation (e.g., Panno et al., 2017). We argue that this, in turn, could be associated with a more self-aware, healthier, more sustainable and environmentally aware food lifestyle (see also Fischer et al., 2017). This idea is consistent also with studies showing a more general association between mindfulness, relations to nature, environmental concern and sustainable lifestyles (e.g., Brown and Kasser, 2005; Fabjański and Brymer, 2017; Panno et al., 2018). The findings from a recent multi-national EU-funded research project (the GLAMURS project^2^), exploring the role of different sustainable initiatives (such as, for example, urban organic agricultural cooperatives), are also supporting this assumption (e.g., Frantzeskaki et al., 2016; Fischer et al., 2018).

## Concluding Remarks

According to the literature we have briefly reviewed, the development of evidence-based policies in the domain of more sustainable food choices could be based on a combination of cultural and education interventions with urban planning management and transformation. Health and environmental issues connected to food consumption and lifestyle are a relevant phenomenon in the western industrialized Western world and a worrying trend in many developing countries. Furthermore, beside genetics, socioeconomic status and differential exposure to environmental features are key factors in overweight and cardiovascular disease risks. This makes it relevant to consider the cultural aspects of an individual life in order to design prevention programs and education interventions. The cultural values and representations attached to food and health should be the target of community interventions, aiming to improve people’s healthy food choices and lifestyles, also through the provision of more restorative and walkable urban settings, to promote physical activity for individuals and communities. Different cultures might differ not only in the way food is produced, prepared and consumed, but also in the attitudes toward the relationship between food, health and pleasure, and in the ways through which food is acquired, prepared, processed and consumed in everyday physical spaces. People’s choice to buy or not, and to eat or not a certain food, is necessarily implicating identity processes and signaling one’s own stance in the social, political and ideological arenas, but it might also be related to features of the physical environment, such as those that afford more sustainable, mindful and self-aware consumption patterns. Within this context, social relationships in the symbolic and physical correlates of food consumption are also relevant. The individual access to the values, habits and places of her/his culture is allowed by the socialization processes that occur via the interpersonal relationships beginning at infancy. We learn what, when, how and where to eat by participating in the eating practices of our family, our community, and other social groups to which we belong, from which we acquire cultural norms and which teach us how to cope with the changes of norms and values. Positive social relationships could have a key role in the food production process too, as an important factor involved in the building of social capital, trust and social support, in order to facilitate small scale agricultural production and equitable markets in the contemporary industrialized metropolis as well as in poorer areas of the world (e.g., Lyon, 2000). In his brilliant and much widely cited psychological account of urban experience, Milgram (1970) identified cognitive and social overloads, accelerated pace of life and diminished social responsibility as distinctive and negative features of urban life. More recently, other authors have provided robust arguments suggesting how urban growth and size is associated with both higher socioeconomic productivity and wealth but also stronger inequalities (Bettencourt et al., 2007; Brelsford et al., 2017). Walkability and restoration opportunities might help to buffer the impact of such negative features, without affecting the positive aspects of increased urbanism. Therefore, one could expect that providing city dwellers with more walkable settings and better restoration opportunities, by positively impacting on citizen’s wellbeing and social relations, could, in turn, promote the diffusion of healthier and more sustainable food consumption patterns, as they contribute to buffering the negative psychological outcomes of urban life.

**FIGURE 1:**
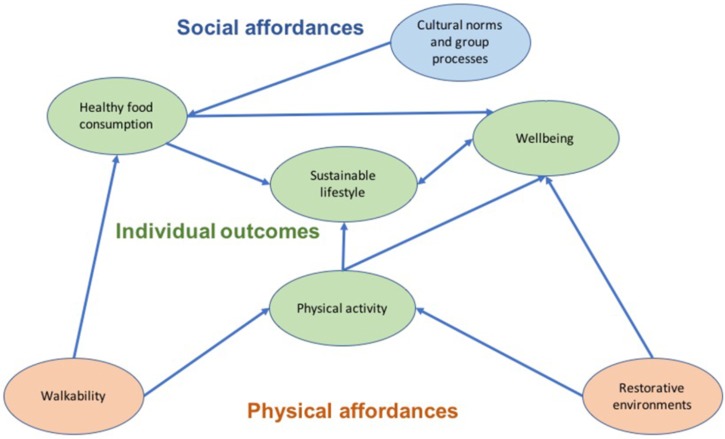
A conceptual model.

Education programs and community interventions on food choices can thus be improved by acting on a multiplicity of factors, ranging from the individual level, to group processes and collective factors, to environmental affordances. Issues of culture, identity and relationships should thus be at the base of targeted communication and intervention programs to achieve desired future scenarios in food choices among the larger public in their everyday settings. Likewise, future research could better investigate the links between people’s health and wellbeing in urban contexts and sustainable lifestyle change. It is indeed commonly assumed, as we have highlighted through the literature reviewed in this paper, that changing lifestyle in a more sustainable direction could have a positive impact on peoples’ wellbeing, for example by eating healthier and more sustainable food. Likewise, empirical studies available to date suggest that promoting outdoor physical activity through more accessible urban green spaces or other nature-based solutions, or through designing more walkable cities and neighborhoods, could also help the promotion of sustainability and wellbeing. An overview of the conceptual relations among all these factors is provided in Figure 1.

In our model, we consider both walkability and restorative environments to be aspects of the physical environment that are likely to promote positive outcomes for the individual, such as more sustainable lifestyles and wellbeing. Based on existing empirical studies, physical activity could be a key mediating mechanism involved in these relations. In addition to that, we argue here that cultural norms and group processes could represent “social affordances” that might also drive an individual’s pursuit of more sustainable lifestyles and wellbeing. A relevant mediating mechanism in this case could be identified in healthy food consumption. Some of the relations that we envisage in our conceptual model have been supported by previous empirical studies (e.g., the relation between restorative environments and wellbeing). However, empirical research is still needed to shed more light on all the relations depicted in Figure 1.

At the same time, future research could also aim to explore whether a reverse pattern is also likely to occur, i.e., increasing wellbeing in currently urbanized society might lead to more sustainable behavior and lifestyles. Some works on the concept of positive environmental psychology (e.g., Corral-Verdugo, 2012) suggest that this is indeed a plausible pattern, but more empirical research is needed on this issue.

In this paper we have provided arguments and reviewed previous empirical studies suggesting links between cultural norms and cultural processes such as migration and acculturation, healthy food consumption patterns, and sustainable lifestyles. Still, more research is needed on the links between cultural processes and positive urban environmental affordances. Since more studies on restorative environments have been conducted in western societies, it would be interesting to further investigate this issue through cross-cultural studies. In this domain, other interesting research questions could also be identified: for example, one might argue that walkable urban settings could be particularly restorative to migrants, as access to green space has been shown to be a powerful tool for tackling social inequalities (e.g., Mitchell and Popham, 2008). Cities can in fact promote sustainable development goals and economic growth but need to undertake the challenge of reducing social inequalities (e.g., Brelsford et al., 2017). Previous studies (e.g., Kyttä et al., 2016) suggest interesting differences in the relations between environmental features and wellbeing across central and peripheral urban settings. Therefore, a challenge for more inclusive urban planning would be to increase walkability, provide more restoration opportunities, and promote access to more healthy food in urban peripheries, where many migrant communities live.

## Author Contributions

GC and SP contributed equally to writing a first draft of the text and shared first authorship. SM revised and rewrote part of the text.

## Conflict of Interest Statement

The authors declare that the research was conducted in the absence of any commercial or financial relationships that could be construed as a potential conflict of interest.

## Footnotes

1 https://www.glopan.org/

2 www.glamurs.eu
